# Purge Haplotigs: allelic contig reassignment for third-gen diploid genome assemblies

**DOI:** 10.1186/s12859-018-2485-7

**Published:** 2018-11-29

**Authors:** Michael J. Roach, Simon A. Schmidt, Anthony R. Borneman

**Affiliations:** The Australian Wine Research Institute, PO Box 197, Glen Osmond, SA 5064 Australia

**Keywords:** Synteny reduction, Redundant contigs, Polymorphic genome

## Abstract

**Background:**

Recent developments in third-gen long read sequencing and diploid-aware assemblers have resulted in the rapid release of numerous reference-quality assemblies for diploid genomes. However, assembly of highly heterozygous genomes is still problematic when regional heterogeneity is so high that haplotype homology is not recognised during assembly. This results in regional duplication rather than consolidation into allelic variants and can cause issues with downstream analysis, for example variant discovery, or haplotype reconstruction using the diploid assembly with unpaired allelic contigs.

**Results:**

A new pipeline—Purge Haplotigs—was developed specifically for third-gen sequencing-based assemblies to automate the reassignment of allelic contigs, and to assist in the manual curation of genome assemblies. The pipeline uses a draft haplotype-fused assembly or a diploid assembly, read alignments, and repeat annotations to identify allelic variants in the primary assembly. The pipeline was tested on a simulated dataset and on four recent diploid (phased) de novo assemblies from third-generation long-read sequencing, and compared with a similar tool. After processing with Purge Haplotigs, haploid assemblies were less duplicated with minimal impact on genome completeness, and diploid assemblies had more pairings of allelic contigs.

**Conclusions:**

Purge Haplotigs improves the haploid and diploid representations of third-gen sequencing based genome assemblies by identifying and reassigning allelic contigs. The implementation is fast and scales well with large genomes, and it is less likely to over-purge repetitive or paralogous elements compared to alignment-only based methods. The software is available at https://bitbucket.org/mroachawri/purge_haplotigs under a permissive MIT licence.

**Electronic supplementary material:**

The online version of this article (10.1186/s12859-018-2485-7) contains supplementary material, which is available to authorized users.

## Background

Recent advances in third-generation single-molecule sequencing have enabled de novo genome assemblies that have extremely high levels of contiguity and completeness [1–3]. Furthermore, recent advances in ‘diploid aware’ genome assemblers have considerably improved the quality of highly heterozygous diploid genome assemblies [4, 5]. Diploid-aware assemblers such as FALCON and Canu are available that will produce a haplotype-fused representation of a diploid genome [4, 6], and some assemblers such as FALCON Unzip and Supernova will go further to produce large phase blocks where both parent alleles are represented separately [4, 7]. For FALCON Unzip assemblies, which are the focus of this study, phasing occurs on the assembly graph to produce ‘primary contigs’ (the haploid assembly) and associated ‘haplotigs’, with the diploid assembly consisting of the union of these primary contigs and secondary haplotigs.

An ideal haploid representation (primary contigs) would consist of one allelic copy of all heterozygous regions in the two haplomes, as well as all hemizygous regions from both haplomes. This ensures that any region in either haplome aligns in its entirety to a single location in the haploid representation. The secondary haplotigs should contain one of the two allelic copies of the heterozygous regions found in both haplomes; in this regard the haplotigs serve as phasing information for the haploid representation.

Regions of very high heterozygosity still present a problem for de novo genome assembly [8–10]. In this situation, once a pair of allelic sequences exceeds a certain threshold of nucleotide diversity, most algorithms will assemble these regions as separate contigs, rather than the expected single haplotype-fused contig [11, 12]. This results in an assembly that is significantly larger than haploid genome size, and the presence of these allelic contigs in a haploid assembly is problematic for downstream analysis [13]. In the case of producing a diploid assembly, while both alleles may be present, steps are still required to identify the allelic contig pairings.

Several tools have attempted to deal with this problem. The HaploMerger2 toolkit [14] and Redundans assembly pipeline [15] were designed to produce haplotype-fused assemblies from short-read sequences. However, the automated removal of contigs based only on alignments of contigs to each other without considering read depth of coverage may lead to repetitive and paralogous contigs being over-purged. Furthermore, resolving the haplotype sequences and producing a phased assembly has proven to be advantageous [16, 17]. Scripts available for use with long-read assemblies include; get_homologs.py, which uses sequence alignments to identify homologues and assist in manual curation [18] and HomolContigsByAnnotation, which uses gene annotations to match syntenic regions [19]. Each has its unique strengths and drawbacks, but both suffer from requiring manual reassignment of contigs by the user.

The aim of this study was to develop a new pipeline that could quickly and automatically identify and reassign allelic contigs specifically in assemblies produced with single-molecule long-read sequencing technology. Purge Haplotigs is designed to be easy to install and requires only three commands to complete. It will work on either the haploid assembly to produce a deduplicated haploid assembly, or on the diploid assembly to produce an improved, deduplicated primary haploid assembly and a more complete secondary haplotig assembly. Finally, the pipeline also produces several outputs designed to assist in the manual inspection and curation of an assembly if desired.

## Implementation

**Fig. 1:**
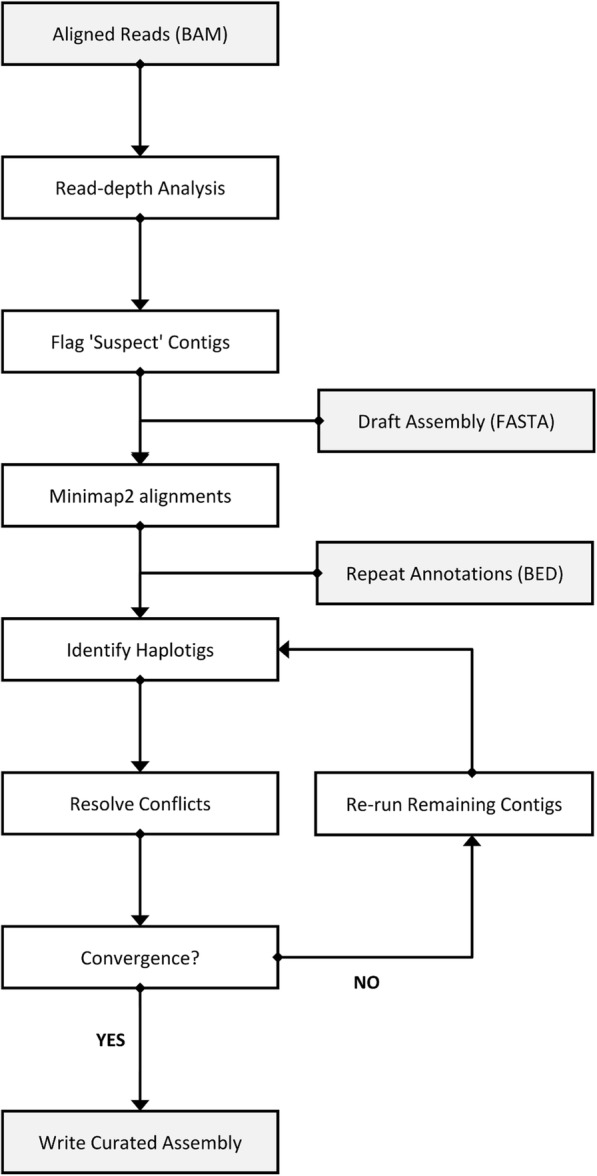
Flow-chart for the Purge Haplotigs pipeline

The Purge Haplotigs pipeline is outlined in Fig. 1. The pipeline requires two input files: a draft assembly in FASTA format, and an alignment file of reads mapped to the assembly in BAM format. The input draft assembly can be either a haploid assembly (e.g. FALCON or CANU) or a diploid assembly (e.g. FALCON Unzip). Repeat annotations can optionally be supplied (in BED format) for improved handling of repeat-rich contigs. For the aligned reads, the pipeline works best when the long-reads that were used for generating the assembly are mapped, but it will also work using short reads. A ‘random best’ alignment should be used for multi-mapping reads and the library should be one that produces an unbiased flat read-coverage.

### Read-depth analysis

The first stage involves a read-depth analysis of the BAM file. A read-depth histogram is initially produced for the assembly. For collapsed haplotype contigs the reads from both alleles will map, whereas if the alleles have assembled as separate contigs the reads will be split over the two contigs, resulting in half the read-depth. This is leveraged to identify contigs that are likely to be haplotigs.

**Fig. 2:**
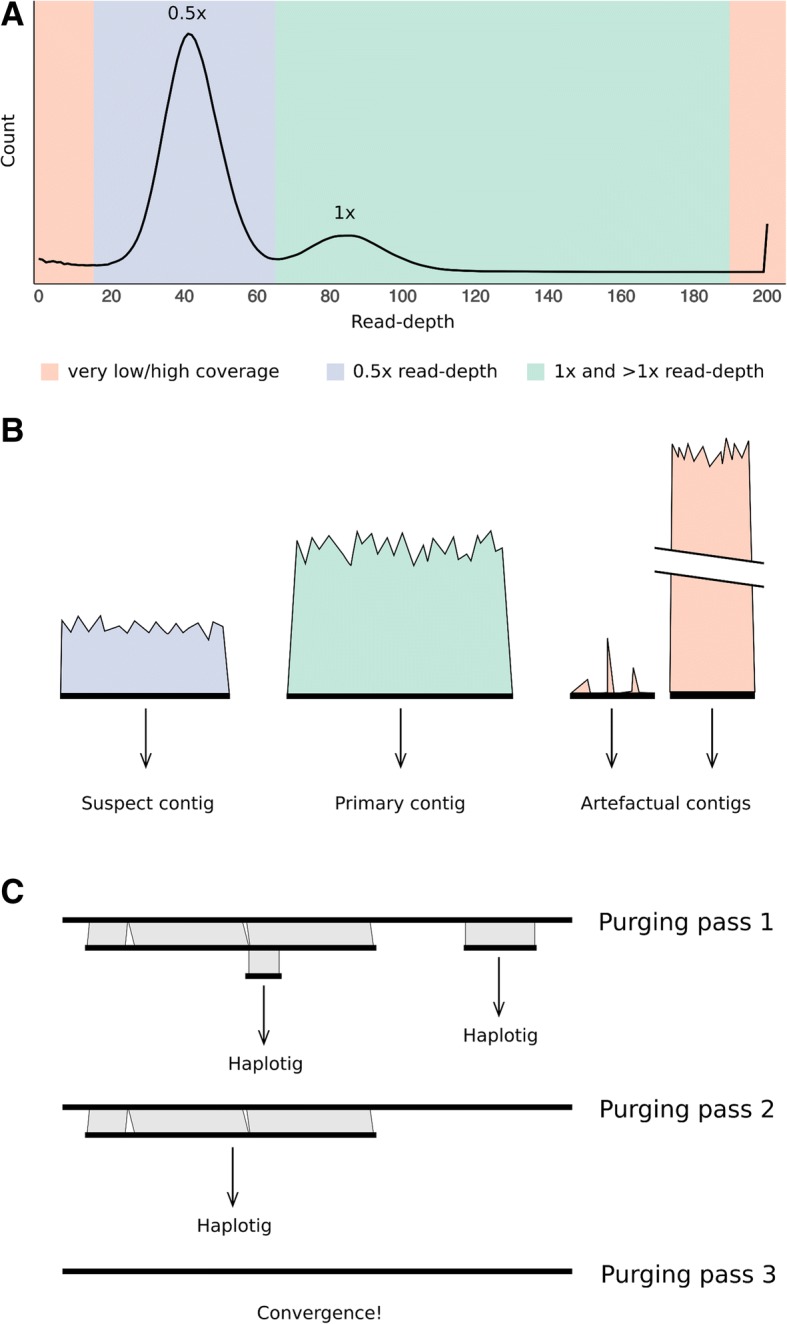
Purge Haplotigs Implementation. **a** Genome-wide read-depth histogram. Bimodal distribution results from the presence of allelic contigs (0.5× coverage) and haplotype-fused contigs (1× coverage). This example histogram uses a low cutoff of 15, a midpoint of 65, and a high cutoff of 190—required in the second step for Purge Haplotigs. **b** Read-depth of individual contigs is used to identify contigs that are suspected to be duplicated. Contigs with abnormally low or high coverage are optionally removed from the assembly. **c** Contigs are aligned and haplotigs are removed iteratively

For a haploid assembly, a bimodal distribution should be observed if duplication has occurred (Fig. 2a). The 0.5× read-depth peak results from the duplicated regions and the 1× read-depth peak results from regions that are properly haplotype-fused. For a diploid assembly, as the entire assembly should be duplicated, the 1× peak may only be very small or not visible at all. The user chooses three cut-offs to capture the two peaks and the pipeline then calculates a breakdown of the read-depth proportions for each contig (Fig. 2b). Contigs with a low proportion of bases within the 1× read-depth range (by default ≤ 80%) are flagged for further analysis. For a diploid assembly, as both haplotypes should be present, most of the contigs would be expected to be flagged for further analysis. Contigs with a high proportion of bases (by default ≥ 80%) at an abnormally low read-depth are likely to be assembly artefacts, and at an abnormally high read-depth are likely to be collapsed repeats or organelle contigs. These contigs optionally can be separated from the rest of the assembly.

### Identification and assignment of homologous sequences

Contigs that were flagged for further analysis according to read-depth are then subject to sequence alignment to attempt to identify synteny with its allelic companion contig. All flagged contigs therefore undergo a Minimap2 search [20] against the entire assembly to identify discrete regions of nucleotide similarity. Using these data Purge Haplotigs ranks the hit contigs for each flagged contig by total number of matching bases. It then calculates both the total portion of the flagged contig that aligns at least once (alignment score) and the sum of all alignments (max match score) between the flagged contig and its two best hit contigs. If repeat annotations have been supplied, alignments to repetitive regions will be ignored when calculating the alignment and max match scores. Contigs with an alignment score greater than the cut-off (by default ≥ 70%) are marked for reassignment as haplotigs. Contigs marked for reassignment with a max match score greater than the cut-off (by default ≥ 250%) are further labelled as repetitive to highlight potential problematic contigs such as collapsed repeats or low-complexity regions.

Conflicts may arise where haplotigs are nested, overlap, or are comprised of mostly repetitive sequence. This can cause individual contigs to be both marked for reassignment and used as a reference for marking another contig for reassignment (Fig. 2c). If a contig and its best hit are both marked for reassignment then only the shorter contig will be reassigned and the longer contig will need to be reanalysed. For this reason, the hit contig identification, alignment scoring, conflict resolution and contig reassignment steps occur iteratively until no more conflicts occur and no more contigs meet the conditions for reassignment as a haplotig.

### Outputs

Purge Haplotigs produces three FASTA format files for the curated assembly: the curated contigs, the contigs reassigned as haplotigs, and the abnormal coverage contigs reassigned as artefacts. If the original input were a draft haploid assembly, then the curated contigs would represent the haploid assembly. Alternatively, if the original input were a draft diploid assembly then the curated contigs represent the haploid assembly, while the revised diploid assembly would consist of the combination of both the curated primary contigs and the reassigned haplotigs. The revised diploid assembly is identical to the draft diploid assembly with the exceptions that allelic contigs are paired and abnormal coverage contigs are optionally removed.

In addition to the FASTA output, Purge Haplotigs also produces several metrics to aid in the manual assessment of the automatic contig assignment function, including the optional production of dotplots juxtaposed with read-depth tracks for each reassigned and ambiguous contig. A data table is produced which lists each contig reassignment and includes both the alignment and max match scores. Finally, a text file is produced to show the contig purging order for the situations in which conflicts were detected. This last file is particularly useful for producing dotplots for visualising haplotig nesting and overlaps, as well as assessing any potential over-purging (for instance if the threshold for reassignment were set too low).

### Limitations

Purge Haplotigs has currently only been tested against diploid genomes. It should be noted that haplotype switching often occurs in the FALCON Unzip primary contigs between neighbouring phase blocks. Breaks in phasing may occur due to a large distance between consecutive variants and longer-range connectivity information is generally needed to completely reconstruct the two haplomes. As such Purge Haplotigs cannot resolve haplotype switching. Instead, it will only attempt to identify contigs that are allelic and produce a deduplicated representation of the genome.

## Results and discussion

### Materials and methods used for pipeline evaluation

The Purge Haplotigs pipeline was first validated using a synthetic dataset (Additional file 1). However, to fully investigate the practical aspects and impact of synteny reduction, Purge Haplotigs was also tested on four draft assemblies produced by FALCON Unzip. Assemblies for *Arabidopsis thaliana* (Cvi-0 × Col-0), *Clavicorona pyxidata* (a coral fungus), and *Vitis vinifera L. Cv.* Cabernet Sauvignon (grapevine) were sourced from Chin, Peluso [4], and a fourth assembly for *Taeniopygia guttata* (Zebra finch) genome was sourced from Korlach, Gedman [5]. Inbred Col-0 and Cvi-0 assemblies were also sourced from Chin, Peluso [4] for analysis of this *Arabidopsis* trio. For each assembly, alignment files which consisted of PacBio RS II SMRT subreads mapped to each of the draft diploid assemblies, were generously provided by Pacific Biosciences.

Purge Haplotigs and Redundans were tested using a 16-core Intel® Xeon® E5-2670 based workstation with 64 GB of available RAM, running Ubuntu 16.04 LTS. Pipelines were instructed to utilise all 32 threads, except for the Purge Haplotigs ‘purge’ stages for *V. vinifera* (16 threads), and *T. guttata* (10 threads) due to RAM constraints. Repeat annotations were produced with RepeatMasker [21] using RepBase version 2017-01-27 [22]. Assembly metrics were calculated using Quast v4.5 [23]. Genome completeness, duplication, and fragmentation were predicted using Benchmarking Universal Single-Copy Orthologs (BUSCOs) using the pipeline of the same name—BUSCO v3.0.1 [24]. Phasing coverage was calculated and visualised from whole genome alignments, and genome sequence comparisons were conducted using the MUMmer package v4.0.0 [25]. Haploid assemblies were assessed for uniform read depth of coverage and heterozygous SNP detection using short read data. Suitable Illumina paired-end (PE) short reads were publicly available from the Sequence Read Archive (SRA) for *A. thaliana* Col-0 × Cvi-0 (SRA accessions: SRR3703081, SRR3703082, SRR3703105), *C. pyxidata* (SRA accession: SRR1800147), and *T. guttata* (SRA accession: ERR1013157). PE reads were downloaded and mapped using BWA-MEM v0.7.12 [26] to the draft and curated haploid assemblies. Heterozygous SNPs were called using VarScan v2.3.9 [27], and read-depth and SNP density were analysed using BEDTools v2.25.0 [28]. The SNP density and read-depth histograms were visualised as Circos plots [29]. Detailed workflows for processing with Purge Haplotigs and subsequent analysis are available in Additional file 1.

### Resource usage

Total runtime and peak RAM usage of Redundans and Purge Haplotigs against all four genomes are reported in Table 1. Purge Haplotigs is optimised for thread utilisation. As such, it was able to process all four genomes quickly with runtime scaling well with genome size. Peak RAM usage for Purge Haplotigs occurs during the parallel Minimap2 alignments. For *A. thaliana*, peak RAM was just under 1 GB per parallel Minimap2 alignment. For *V. vinifera* and *T. guttata*, the parallel Minimap2 alignments had to be reduced to 16 and 10 respectively as the peak RAM was much higher for these larger genomes (approximately 3 GB and 10 GB per parallel Minimap2 alignment respectively).

**Table 1:** Pipeline runtimes and peak RAM usage for Redundans and Purge Haplotigs

	Diploid genome size (Mbp)	Redundans		Purge Haplotigs	
		Runtime (hh:mm:ss)	Peak RAM (GB)	Runtime (hh:mm:ss)	Peak RAM (GB)
*C. pyxidata*	65.4	00:01:54	1	00:01:04	10
*A. thaliana*	245	00:35:56	1	00:04:17	30
*V. vinifera*	959	15:48:27	6	00:34:40	47
*T. guttata*	1983	06:14:06	6	01:04:51	60

### Purge Haplotigs effectively optimises Arabidopsis model assembly

In order to quantify the effectiveness of the Purge Haplotigs pipeline, its performance was assessed using genome assemblies from a previously-established trio of *A. thaliana* isolates [4]. These consist of a draft diploid assembly of a heterozygous F1 line as well as highly contiguous and accurate assemblies of both homozygous parents (Col-0 and its most divergent relative Cvi-0). Furthermore, the chromosome-resolved assembly for Col-0 (TAIR10) was also available to enable detailed chromosome-scale comparisons [30].

The ideal haploid representation of the F1 cross of Col-0 and Cvi-0 should consist of one allelic copy of all common regions between the Col-0 and Cvi-0 parent genomes, as well as all hemizygous regions from both parent genomes. The haplotigs should consist of the other allelic copies of the Col-0 and Cvi-0 common regions. The Col-0 and Cvi-0 parent genomes should therefore align in their entirety to the haploid representation (primary contigs), and as completely as possible to the haplotigs. To determine if Purge Haplotigs provided improvements to this metric, the draft assembly and the Purge Haplotigs- and Redundans-processed assemblies were compared with the two parent genome assemblies.

The coverage of the Col-0 and Cvi-0 parent genomes by the draft primary contigs was high at 97.9% for both (Table 2). However, the draft haplotigs only aligned to an average 87.6% of the parent genomes. The Purge Haplotigs-processed primary contigs showed a 1% decrease in coverage of the parent genomes, indicating that some over-purging is occurring. However, there was an average 94.4% coverage of the parent genomes by haplotigs. This increase is much higher than the drop in coverage and suggests a strong enrichment for deduplication over reduction in genome completeness.

**Table 2:** Whole genome alignments of the *A. thaliana* draft and processed assemblies to the homozygous parent genomes

	Col-0 alignments		Cvi-0 alignments	
	Coverage (% Col-0 length)	Identity (%)	Coverage (% Cvi-0 length)	Identity (%)
FALCON Unzip				
- Primary contigs	97.9	98.7	97.9	98.6
- Haplotigs	87.5	98.5	87.7	98.6
Purge Haplotigs				
- Primary contigs	96.9	98.8	96.6	98.6
- Haplotigs	94.2	98.5	94.9	98.6
Redundans				
- Reduced contigs	95.5	98.8	94.6	98.5

**Fig. 3:**
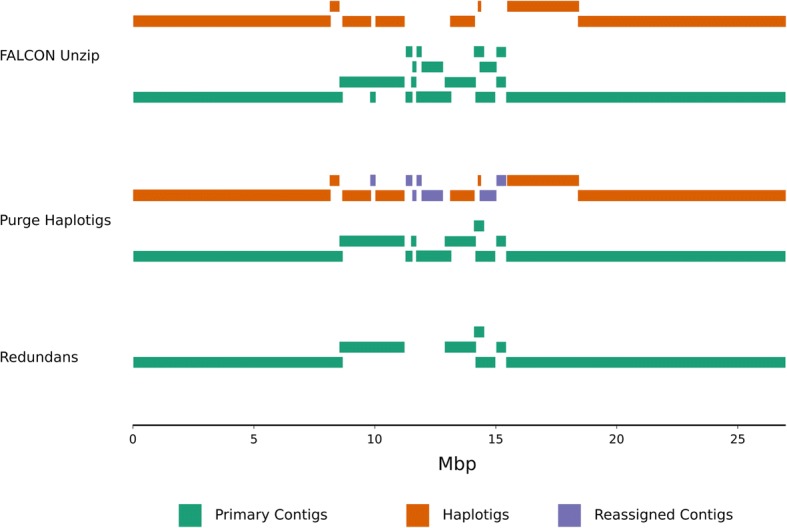
Alignments of contigs to Chromosome 5 of the TAIR10 (Col-0) reference genome. Alignments for the draft FALCON Unzip, and the Purge Haplotigs- and Redundans-processed assemblies are shown as stacked horizontal bars and are juxtaposed vertically

Purge Haplotigs fills gaps in the haplotig tiling path using duplicated allelic contigs from the primary contig pool. This solves both the problem of duplication in the haploid representation (primary contigs) as well as the problem of phasing gaps in the haplotigs. In order to visualise this on a chromosome-scale, the draft assembly and the Purge Haplotigs- and Redundans-processed assemblies were aligned to the chromosome-resolved TAIR10 (Col-0) reference assembly. Chromosome 5 was selected to highlight the utility of contig reassignment, and the contig alignments for this chromosome are shown in Fig. 3 as stacked bars. For the draft assembly, there are two large gaps in the coverage of haplotig alignments to Chromosome 5; this coincides with duplicate alignments in the primary contigs. Primary contig duplication is reduced and haplotig coverage of the reference genome is greatly improved following the reassignment of duplicated contigs by Purge Haplotigs.

### Deduplication reduces assembly size

Once it had been established that Purge Haplotigs was able to accurately deduplicate the *A. thaliana* dataset, the assembly statistics were examined. As mentioned, haploid assemblies contaminated by allelic contigs can be significantly larger than the haploid genome size. The draft FALCON Unzip haploid assembly for *A. thaliana* was 140 Mb, much larger than the current TAIR10 (Col-0) reference genome of 119 Mb [30]. The haploid assembly size was reduced to 126 Mbp by Purge Haplotigs, placing it closer to the Col-0 haploid size (Table 3). Furthermore, the reduction in haploid genome size is almost entirely attributed to the identification and reassignment of haplotigs, rather than the removal of artefactual contigs which only accounted for 1.7 Mbp of the assembly. The Redundans-processed assembly was reduced to 119 Mbp; while this mirrors the haploid genome size, the haploid representation of the heterozygous genome is expected to be larger if it includes the hemizygous regions from both parents. For the other assemblies in this case study, the haploid sizes were also reduced by between 4.1% (*C. pyxidata*) and 12.0% (*V. vinifera*) (Additional file 2) after processing with Purge Haplotigs.

**Table 3:** Assembly statistics for draft FALCON Unzip, Redundans-processed and Purge Haplotigs-processed *A. thaliana* assemblies

	Haploid Assemblies (Primary contigs)			Haplotigs		Artefacts
	FALCON Unzip	Purge Haplotigs	Redundans	FALCON Unzip	Purge Haplotigs	Purge Haplotigs
Contigs	172	120	93	248	200	100
Contigs > = 1000 bp	171	119	92	248	200	100
Contigs > = 10,000 bp	171	108	92	214	200	77
Total length (Mbp)	140.0	125.6	119.2	104.9	117.7	1.740
GC (%)	36.7	36.7	36.6	36.1	36.1	38.5
N50 (Mbp)	7.96	7.98	7.98	6.92	4.63	0.0199

### Genome completeness is unaffected by contig reassignment

While the Arabidopsis trio was useful in evaluating the performance of Purge Haplotigs for the *A. thaliana* assembly, an orthologous method was required for the other assemblies in this case study. BUSCOs are sets of known gene orthologs that are predicted to be present as a single copy in a genome. They are used extensively for estimating the completeness, duplication and fragmentation of genome assemblies [1, 4, 31, 32]. The primary contigs and haplotigs of the draft FACLON Unzip and the Purge Haplotigs-processed assemblies were therefore evaluated using the BUSCO pipeline, as were the Redundans-processed (haploid) assemblies (*A. thaliana* Table 4, Additional file 2). The TAIR10 and Cvi-0 assemblies are also included for comparison in Additional file 2. Finally, the artefact contigs removed by Purge Haplotigs were also assessed to determine if the removal of these contigs was detrimental to the predicted completeness of the genome assemblies.

**Table 4:** BUSCO statistics for draft FALCON Unzip, Redundans-processed, and Purge Haplotigs-processed *A. thaliana* assemblies

	Haploid Assemblies (Primary contigs)						Haplotigs				Artefacts	
	FALCON Unzip		Purge Haplotigs		Redundans		FALCON Unzip		Purge Haplotigs		Purge Haplotigs	
	#	%	#	%	#	%	#	%	#	%	#	%
Total BUSCOs	1440	100.0	1440	100.0	1440	100.0	1440	100.0	1440	100.0	1440	100.0
Complete BUSCOs	1413	98.1	1407	97.7	1407	97.7	1342	93.2	1400	97.2	17	1.2
- single-copy	1324	91.9	1377	95.6	1378	95.7	1313	91.2	1372	95.3	17	1.2
- duplicated	89	6.2	30	2.1	29	2.0	29	2.0	28	1.9	0	0.0
Fragmented BUSCOs	5	0.3	8	0.6	8	0.6	5	0.3	5	0.3	3	0.2
Missing BUSCOs	22	1.5	25	1.7	25	1.7	93	6.5	35	2.4	1420	98.6

The Purge Haplotigs haploid assemblies (primary contigs) contained between 39% (*C. pyxidata*) and 66% (*A. thaliana*) fewer duplicated BUSCOs compared to the draft assemblies, and contained similar total BUSCOs, ranging from 0.4% fewer (*A. thaliana*) to 3.6% more (*V. vinifera*) BUSCOs. An increase in total BUSCOs found in the primary contigs can occur where a FALCON Unzip haplotig is longer than its primary contig due to the inclusion of large structural variants. These large structural variants may contain extra BUSCOs. Purge Haplotigs will always keep the longer contig which results in an occasional ‘swapping’ of primary contigs and haplotigs compared to the draft FALCON Unzip assembly. When comparing Purge Haplotigs to Redundans, the Purge Haplotigs haploid assemblies contained between 3.4% more (*A. thaliana*) and 70.8% fewer (*C. pyxidata*) duplicated BUSCOs, and there was very little difference in the number of complete BUSCOs found.

The haplotigs from the draft assemblies and the Purge Haplotigs-processed assemblies were compared. The processed haplotigs contained between 7.1% (*C. pyxidata*) and 62.4% (*A. thaliana*) fewer missing BUSCOs. This suggests that the haplotigs are themselves more complete representations of their genomes after processing with Purge Haplotigs. This is consistent with the findings of the genome comparisons using the Arabidopsis trio. Finally, there were only between 0.2% (*C. pyxidata*) and 1.4% (*V. vinifera*) of BUSCOs found in the Purge Haplotigs artefactual contigs, and in all cases, all BUSCOs identified were confirmed to be copies that were also present in the assembly’s remaining contigs.

### Contig reassignment improves haplotig coverage

**Fig. 4:**
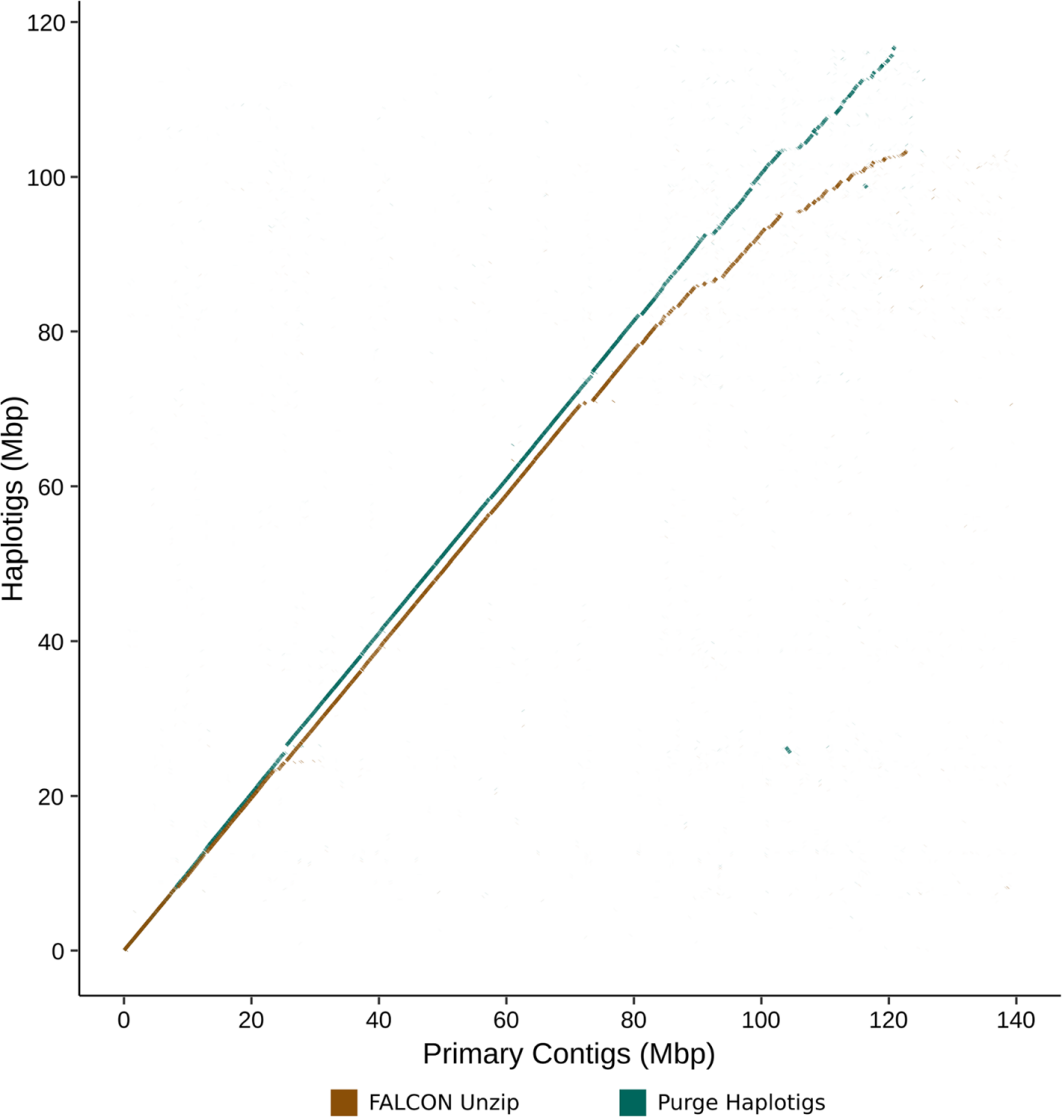
Dotplots for *Arabidopsis thaliana* assemblies. Haplotigs were aligned to primary contigs, total coverage of primary contigs by haplotigs was calculated, dotplots for one-to-one best alignments are shown. There was 78.7 and 90.5% coverage of primary contigs by haplotigs for the draft FALCON Unzip and the Purge Haplotigs-processed assemblies respectively. Vertical gaps correspond to sequence in haplotigs that is not present in the primary contigs, and horizontal gaps correspond to sequence in the primary contigs not present in the haplotigs

Proper identification of allelic contig pairs results in improved phasing coverage of diploid assemblies. This is shown in Fig. 3 for *A. thaliana*. To assess if Purge Haplotigs provided improvements to this metric, pairwise alignments were performed between the primary contigs and haplotigs for both the draft and Purge Haplotigs-processed assemblies. The total coverage of primary contigs by haplotigs was calculated and visualised (*A. thaliana* Fig. 4; Additional file 3). Coverage of primary contigs by haplotigs increased for all four assemblies. For the *C. pyxidata* and *T. guttata* assemblies the phasing coverage increased by 2.5 and 5.6% respectively. The two plant assemblies—which had higher predicted duplication—showed larger increases in phasing coverage of 11.8 and 11.3% for *A. thaliana* and *V. vinifera* respectively.

### Genome deduplication improves SNP detection

**Fig. 5:**
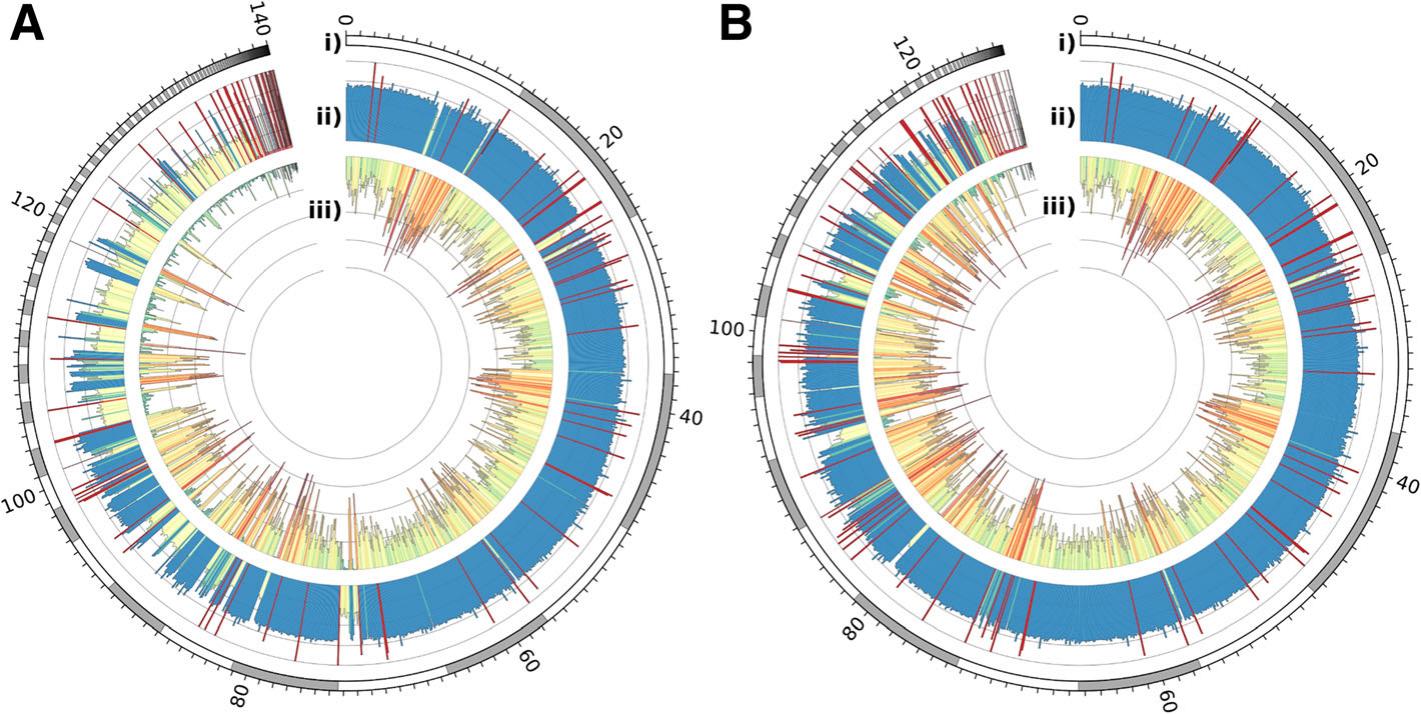
Circos plots for *Arabidopsis thaliana* haploid assemblies. Illumina PE reads were mapped, and heterozygous SNPs were called for the draft FALCON Unzip assembly (**a**) and the assembly curated with Purge Haplotigs (**b**). The tracks shown in the circos plots are: Contigs (ordered by length) (i), Read-depth histogram (reads per genome window; blue: median read-depth, yellow: half read-depth, red: very low/high read-depth) (ii), and SNP density (SNPs per genome window; blue: low SNP density, red: high SNP density) (iii). There were 577.0, 667.0, and 665.1 thousand filtered heterozygous SNP calls for the draft, Purge Hapltogs-processed, and Redundans-processed assemblies respectively

As mentioned previously, the erroneous presence of both allelic contigs in a haploid assembly results in the presence of mapped regions displaying half the average read-depth and few (if any) heterozygous variant calls relative to the rest of the genome. To determine if the use of short-reads for genomic analysis was improved after processing, combined read-depth and heterozygous SNP density plots were generated for the draft assemblies and the Purge Haplotigs-processed assemblies of *A. thaliana*, *C. pyxidata*, and *T. guttata*, based upon the results from mapping illumina PE short-read data to the haploid assemblies. Heterozygous SNPs were stringently filtered to only consider regions with single-copy read-depth (i.e. within the 1× peak in Fig. 2a). There were between 2.7% (*T. guttata*) and 15.6% (*A. thaliana*) more heterozygous SNPs called from the Purge Haplotigs-processed assembly compared to the draft FALCON Unzip assembly (*A. thaliana* Fig. 5; Additional file 4). Furthermore, there were between 0.3% (*A. thaliana*) and 21.1% (*C. pyxidata*) more SNPs called in the Purge Haplotigs-processed assembly compared to the Redundans-processed assembly.

## Conclusions

Purge Haplotigs is an effective tool for the early stages of curating highly heterozygous genome assemblies produced from third-generation long read sequencing. Purge Haplotigs is fast with runtime scaling well with genome size. It can produce a mostly deduplicated haploid representation of a genome which is important for downstream analysis such as variant discovery. Purge Haplotigs can also generate an improved diploid representation of a genome with more allelic contigs identified and properly paired. This is particularly important for diploid assemblies, for instance if attempting to reconstruct parent haplomes.

## Availability and requirements

**Project name:** Purge Haplotigs

**Project home page:** https://bitbucket.org/mroachawri/purge_haplotigs

**Operating system:** Linux (tested on Ubuntu 16.04 LTS)

**Programming language:** Perl

**Dependencies:** BEDTools, SAMtools, Minimap2, Perl, Rscript (with ggplot2)

**License:** MIT

**Restrictions:** None

## Additional files

Additional file 1: Workflows for Purge Haplotigs and subsequent analysis. (PDF 466 kb)

Additional file 2: Quast and BUSCO analysis results for all assemblies. (XLSX 22 kb)

Additional file 3: Dotplots and coverage for *C. pyxidata*, *V. vinifera L. Cv.* Cabernet Sauvignon, and *T. guttata*. (PDF 728 kb)

Additional file 4: Circos Plots and mapping statistics for *C. pyxidata*, and *T. guttata*. (PDF 3318 kb)

## Abbreviations

BUSCO: The name of the pipeline for detecting BUSCOs
BUSCO(s): Benchmarking Universal Single-Copy Ortholog(s)
PE: Paired End
SRA: Sequence Read Archive

## References

[1] Badouin H, Gouzy J, Grassa CJ, Murat F, Staton SE, Cottret L (2017). The sunflower genome provides insights into oil metabolism, flowering and Asterid evolution. Nature.

[2] Jarvis DE, Ho YS, Lightfoot DJ, Schmöckel SM, Li B, Borm TJA, et al. The genome of *Chenopodium quinoa*. Nature. 2017;542(7641):307–12. 10.1038/nature21370 28178233

[3] Loman NJ, Quick J, Simpson JT. A complete bacterial genome assembled *de novo* using only nanopore sequencing data. Nat Meth. 2015;12(8):733–5. 10.1038/nmeth.3444 26076426

[4] Chin C-S, Peluso P, Sedlazeck FJ, Nattestad M, Concepcion GT, Clum A (2016). Phased diploid genome assembly with single-molecule real-time sequencing. Nat Methods.

[5] Korlach J, Gedman G, Kingan SB, Chin C-S, Howard JT, Audet J-N, et al. De novo PacBio long-read and phased avian genome assemblies correct and add to reference genes generated with intermediate and short reads. GigaScience. 2017;6(10):gix085-gix. 10.1093/gigascience/gix085 PMC5632298 29020750

[6] Koren S, Walenz BP, Berlin K, Miller JR, Bergman NH, Phillippy AM. Canu: scalable and accurate long-read assembly via adaptive *k*-mer weighting and repeat separation. Genome Res. 2017;27(5):722–36. 10.1101/gr.215087.116 PMC5411767 28298431

[7] Weisenfeld NI, Kumar V, Shah P, Church DM, Jaffe DB (2017). Direct determination of diploid genome sequences. Genome Res.

[8] Kajitani R, Toshimoto K, Noguchi H, Toyoda A, Ogura Y, Okuno M, et al. Efficient de novo assembly of highly heterozygous genomes from whole-genome shotgun short reads. Genome Res. 2014;24(8):1384–95. 10.1101/gr.170720.113 PMC4120091 24755901

[9] Safonova Y, Bankevich A, Pevzner PA (2015). dipSPAdes: assembler for highly polymorphic diploid genomes. J Comput Biol.

[10] Vinson JP, Jaffe DB, O'Neill K, Karlsson EK, Stange-Thomann N, Anderson S, et al. Assembly of polymorphic genomes: algorithms and application to* Ciona savignyi*. Genome Res. 2005;15(8):1127–35. 10.1101/gr.3722605 PMC1182225 16077012

[11] Pryszcz LP, Németh T, Gácser A, Gabaldón T. Genome comparison of *Candida orthopsilosis* clinical strains reveals the existence of hybrids between two distinct subspecies. Genome Biol Evol. 2014;6(5):1069–78. 10.1093/gbe/evu082 PMC4040990 24747362

[12] Small KS, Brudno M, Hill MM, Sidow A. A haplome alignment and reference sequence of the highly polymorphic *Ciona savignyi* genome. Genome Biol. 2007;8(3):R41. 10.1186/gb-2007-8-3-r41 PMC1868934 17374142

[13] Olson ND, Lund SP, Colman RE, Foster JT, Sahl JW, Schupp JM (2015). Best practices for evaluating single nucleotide variant calling methods for microbial genomics. Front Genet.

[14] Huang S, Kang M, Xu A (2017). HaploMerger2: rebuilding both haploid sub-assemblies from high-heterozygosity diploid genome assembly. Bioinformatics.

[15] Pryszcz LP, Gabaldon T (2016). Redundans: an assembly pipeline for highly heterozygous genomes. Nucleic Acids Res.

[16] Schwessinger B, Sperschneider J, Cuddy WS, Garnica DP, Miller ME, Taylor JM, et al. A Near-Complete Haplotype-Phased Genome of the Dikaryotic Wheat Stripe Rust Fungus* Puccinia striiformis* f. sp. *tritici *Reveals High Interhaplotype Diversity. mBio. 2018;9(1):e02275–17. 10.1128/mBio.02275-17 PMC5821087 29463659

[17] VanBuren R, Wai CM, Ou S, Pardo J, Bryant D, Jiang N, et al. Extreme haplotype variation in the desiccation-tolerant clubmoss *Selaginella lepidophylla*. Nat Commun. 2018;9(1):13. 10.1038/s41467-017-02546-5 PMC5750206 29296019

[18] Concepcion G. get_homologs.py 2016 [Available from: https://github.com/PacificBiosciences/apps-scripts.

[19] Kingan S. HomolContigsByAnnotation 2016 [Available from: https://github.com/skingan/HomolContigsByAnnotation.

[20] Li H (2018). Minimap2: pairwise alignment for nucleotide sequences. Bioinformatics.

[21] Smit A, Hubley R, Green P. RepeatMasker Open-4.0. 2013-2015 [Available from: http://www.repeatmasker.org.

[22] Bao W, Kojima KK, Kohany O (2015). Repbase update, a database of repetitive elements in eukaryotic genomes. Mob DNA.

[23] Gurevich A, Saveliev V, Vyahhi N, Tesler G (2013). QUAST: quality assessment tool for genome assemblies. Bioinformatics.

[24] Simão FA, Waterhouse RM, Ioannidis P, Kriventseva EV, Zdobnov EM (2015). BUSCO: assessing genome assembly and annotation completeness with single-copy orthologs. Bioinformatics.

[25] Kurtz S, Phillippy A, Delcher AL, Smoot M, Shumway M, Antonescu C (2004). Versatile and open software for comparing large genomes. Genome Biol.

[26] Li H (2013). Aligning sequence reads, clone sequences and assembly contigs with BWA-MEM. ARXIV.

[27] Koboldt DC, Zhang Q, Larson DE, Shen D, McLellan MD, Lin L (2012). VarScan 2: somatic mutation and copy number alteration discovery in cancer by exome sequencing. Genome Res.

[28] Quinlan AR, Hall IM (2010). BEDTools: a flexible suite of utilities for comparing genomic features. Bioinformatics.

[29] Krzywinski MI, Schein JE, Birol I, Connors J, Gascoyne R, Horsman D (2009). Circos: An information aesthetic for comparative genomics. Genome Research.

[30] Lamesch P, Berardini TZ, Li D, Swarbreck D, Wilks C, Sasidharan R (2012). The Arabidopsis information resource (TAIR): improved gene annotation and new tools. Nucleic Acids Res.

[31] Bickhart DM, Rosen BD, Koren S, Sayre BL, Hastie AR, Chan S, et al. Single-molecule sequencing and chromatin conformation capture enable *de novo* reference assembly of the domestic goat genome. Nat Genet. 2017;49:643. 10.1038/ng.3802 PMC5909822 28263316

[32] Daccord N, Celton J-M, Linsmith G, Becker C, Choisne N, Schijlen E, et al. High-quality *de novo* assembly of the apple genome and methylome dynamics of early fruit development. Nat Genet. 2017;49:1099. 10.1038/ng.3886 28581499

