# Supplementary material for: Purge Haplotigs: allelic contig reassignment for third-gen diploid genome assemblies
Source: BMC Bioinformatics. 2018 Nov 29;19:460. doi: 10.1186/s12859-018-2485-7 (PMC6267036; doi:10.1186/s12859-018-2485-7)
Supplement: Supplementary file 1 — Workflows for Purge Haplotigs and subsequent analysis. (PDF 466 kb) [file 12859_2018_2485_MOESM1_ESM.pdf]

Workflows for Purge Haplotigs and Analysis

Purge Haplotigs Synthetic Dataset

The validation run on an earlier version of the pipeline, using a simulated genome and simulated PacBio subreads is archived here: https://doi.org/10.5281/zenodo.1042847. The current version of Purge Haplotigs (commit: afc943c) includes the simulation dataset which can be run at any time using the command:

```
purge_haplotigs test
```

Purge Haplotigs Case Study: Running Purge Haplotigs

- Preparation: Mapping subreads

```
minimap2 -ax map-pb genome.fa subreads.fasta.gz \
    | samtools view -hF 256 - \
    | samtools sort -@ 8 -m 1G -o aligned.bam -T tmp.ali
```

Purge Haplotigs was run using a bash script for the purposes of logging memory and runtime over the course of all three commands. The cutoffs for the ‘contigcov’ stages were determined ahead of time.

- *Arabidopsis thaliana* purge_haplotigs.sh run script

```
purge_haplotigs readhist -b ../aligned.bam -g ../genome.fa -t 32
purge_haplotigs contigcov -i aligned.bam.gencov -l 10 -m 95 -h 190
purge_haplotigs purge -g ../genome.fa -c coverage_stats.csv \
    -t 32 -r ../repeats.bed
touch purge_haplotigs.done
```

- *Clavicorona pyxidata* purge_haplotigs.sh run script

```
purge_haplotigs readhist -b ../aligned.bam -g ../genome.fa -t 32
purge_haplotigs contigcov -i aligned.bam.gencov -l 10 -m 65 -h 180
purge_haplotigs purge -g ../genome.fa -c coverage_stats.csv \
    -t 32 -r ../repeats.bed
touch purge_haplotigs.done
```

- *Vitis vinifera L. Cv.* Cabernet Sauvignon purge_haplotigs.sh run script

```
purge_haplotigs readhist -b ../aligned.bam -g ../genome.fa -t 32
purge_haplotigs contigcov -i aligned.bam.gencov -l 10 -m 90 -h 190
purge_haplotigs purge -g ../genome.fa -c coverage_stats.csv \
    -t 16 -r ../repeats.bed
touch purge_haplotigs.done
```

- *Taeniopygia guttata* purge_haplotigs.sh run script

```
purge_haplotigs readhist -b ../aligned.bam -g ../genome.fa -t 32
purge_haplotigs contigcov -i aligned.bam.gencov -l 10 -m 70 -h 190
purge_haplotigs purge -g ../genome.fa -c coverage_stats.csv \
    -t 10 -r ../repeats.bed
touch purge_haplotigs.done
```

- Memory logging script

```
While ! [[ -e purge_haplotigs.done ]]
  do
    free -g | awk '/Mem:/{print $3}' >> mem.log
    sleep 1
done
```

- CPU logging script

```
While ! [[ -e purge_haplotigs.done ]]
  do
    mpstat 1 1 | awk '/Average/{print $3}' >> cpu.log
    sleep 1
done
```

- Runtime logging

```
time ( bash purge_haploitgs.sh 2> purge_haplotigs.log ) \
    2> purge_haplotigs.time
```

- Redundans command (same for all assemblies)

```
redundans.py -f ../genome.fa -t 32 --nocleaning --noscaffolding \
  --norearrangements --nogapclosing
```

Purge Haplotigs Case Study: Illumina Paired End Short Read Mapping

Example workflow for the *C. pyxidata* Purge Haploitgs-processed assembly; the same approach was used for all assemblies with the exception of the read-depth cutoffs during SNP filtering.

- Create BWA index

```
bwa index ../purge_haplotisg/curated.fasta
```

- Map and sort

```
bwa mem ../purge_haploitsg/curated.fasta -t 24 \
  SRR1800147_1.fastq.gz SRR1800147_2.fastq.gz \
  | samtools sort -@ 8 -m 1G -o PH.bam -T ali.tmp
```

- Get stats on mapping

```
samtools flagstat PH.bam
```

- Generate a read-depth histogram to determine SNP filtering cutoffs

```
purge_haplotigs readhist -b PH.bam -g ../purge_haplotigs/curated.fasta -t 32
```

- Call variants and filter on-the-fly (heterozygous only, read-depth between 60 and 140 for *C. pyxidata*, 25 and 60 for *A. thaliana* and 20 and 50 for *T. guttata*)

```
samtools mpileup -f ../genome.fasta PH.bam \
  | java -jar Varscan.jar mpileup2snp --p-value 0.001 \
  | perl -e 'while(<>){my@l=split(/\s+/,$_);
  my @d=split(/:/,$l[4]);($l[7]==1)&&($d[1]>60)&&($d[1]<140)&&
  (print $_);}' \
  | gzip - > PH.SNPs.tsv.gz
```

Purge Haplotigs Case Study: Circos Plots

The workflow example and control file examples are available at:
https://bitbucket.org/mroachawri/read_snp_circos_eg

Purge Haplotigs Case Study: BUSCO Analysis

Example for FALCON Unzip primary contigs shown.

- *A. thaliana*

```
python run_BUSCO.py -i ../genome.p-ctg.fasta -o FALCON \
  -l /datasets/embryophyta_odb9/ -m genome -c 32 -sp arabidopsis
```

- *C. pyxidata*

```
python run_BUSCO.py -i ../genome.p-ctg.fasta -o FALCON \
  -l /datasets/basidiomycota_odb9/ -m genome -c 32 -sp coprinus
```

- *V. vinifera L. Cv.* Cabernet Sauvignon

```
python run_BUSCO.py -i ../genome.p-ctg.fasta -o FALCON \
  -l /datasets/embryophyta_odb9/ -m genome -c 32 -sp arabidopsis
```

- *T. guttata*

```
python run_BUSCO.py -i ../genome.p-ctg.fasta -o FALCON \
  -l /datasets/aves_odb9/ -m genome -c 32 -sp human
```

Purge Haplotigs Case Study: MUMmer Alignments, Dotplots, Alignment Coverages

Generic example workflow

- Calculate alignments between a reference (`ref.fa`) and a query (`query.fa`)

```
nucmer -t 32 ref.fa query.fa -p output
```

- Filter alignments (1-to-1 best) for dotplots

```
delta-filter -1 output.delta > output.1delta
```

- To make dotplots, use mummerplot to generate cords for plotting and a png for previewing

```
mumemrplot --fat --png –large output.1delta -p output
```

- convert coords for plotting in Rstudio using ggplot2

```
cat output.fplot output.rplot | sed ‘s/^$/NA\tNA\tNA/’ > output.gpath
```

- To get the alignment statistics

```
dnadiff -d output.delta -p output
cat output.report
```

- To generate coords for plotting stacked contig alignments to chromosomes in Rstudio using ggplot2, filter for best query-to-reference alignments, extract the contig alignments to the chromosome of interest (Chromosome 5), apply a y-axis offset for each chromosome.

```
delta-filter -q output.delta > output.qdelta
show-coords -HT output.qdelta | awk '{print $8"\t"$1"\t"$2"\t"$9}' \
  | grep Chr5 | sort -k2,2n -k3,3n > output.chr5.bed
XOFF=0; for ii in `cat output.chr5.bed \
| awk '$3-$2>20000{print $4}' | sort | uniq`;
do
  grep $ii output.chr5.bed | awk -v xoff=$XOFF '{print
    $2"\t"xoff"\n"$3"\t"xoff"\nNA\tNA"}'; let XOFF++;
done > output.chr5.gpath
```

- The chromosome .gpath files are then loaded into Rstudio for plotting and an SVG file is exported. Horizontal bars are drawn over the alignments and coloured in inkscape.
