# Supplementary material for: Purge Haplotigs: allelic contig reassignment for third-gen diploid genome assemblies
Source: BMC Bioinformatics. 2018 Nov 29;19:460. doi: 10.1186/s12859-018-2485-7 (PMC6267036; doi:10.1186/s12859-018-2485-7)
Supplement: Supplementary file 3 — Dotplots and coverage for C. pyxidata, V. vinifera L. Cv. Cabernet Sauvignon, and T. guttata. (PDF 728 kb) [file 12859_2018_2485_MOESM3_ESM.pdf]

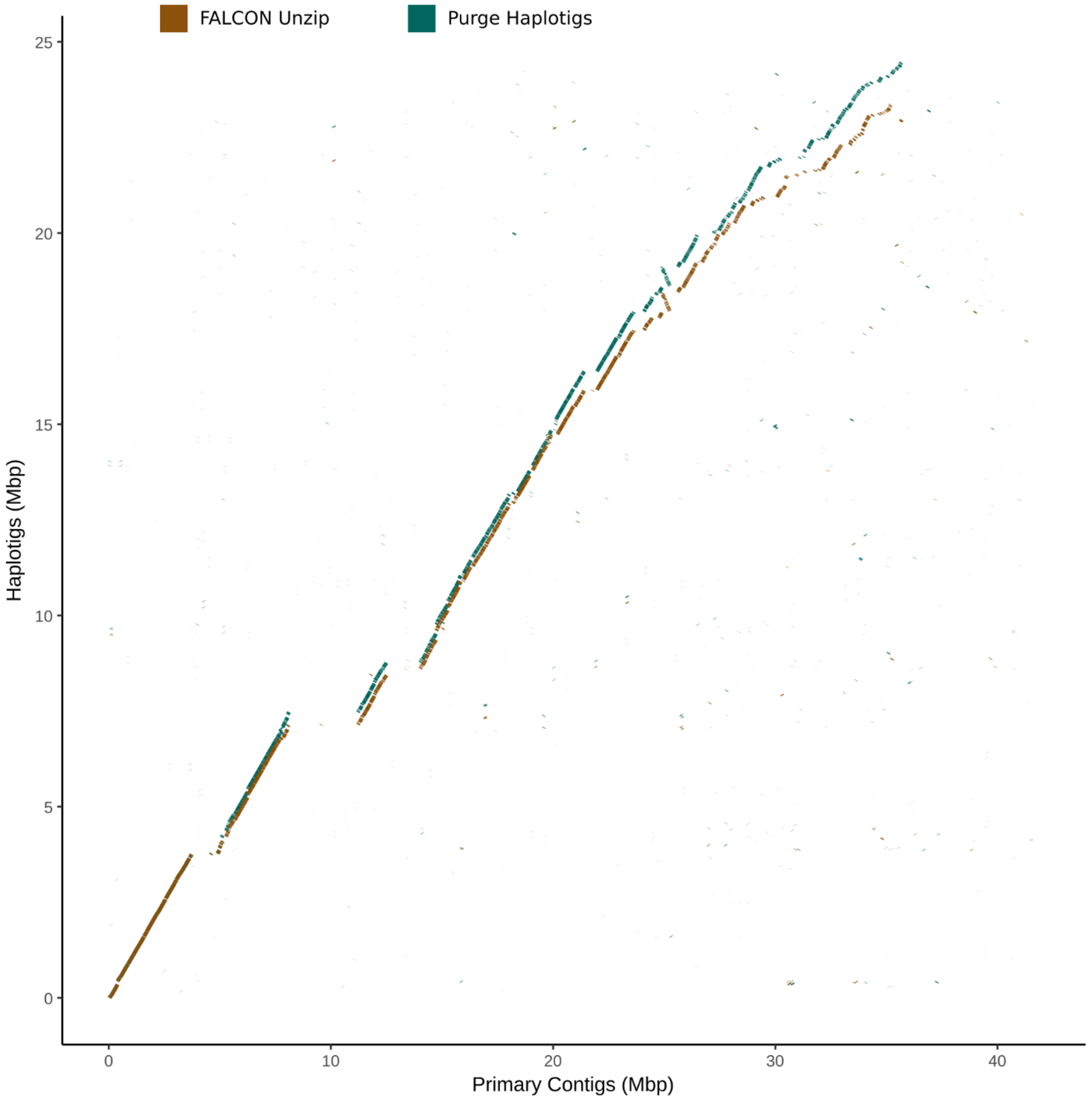

Dotplots for *Clavicorona pyxidata* assemblies. Haplotigs were aligned to primary contigs, total coverage of primary contigs by haplotigs was calculated, dotplots for one-to-one best alignments are shown. There was 56.2 % and 58.7 % coverage of primary contigs by haplotigs for the draft FALCON Unzip and the Purge Haplotigs-processed assemblies respectively. Vertical gaps correspond to sequence in haplotigs that is not present in the primary contigs, and horizontal gaps correspond to sequence in the primary contigs not present in the haplotigs.

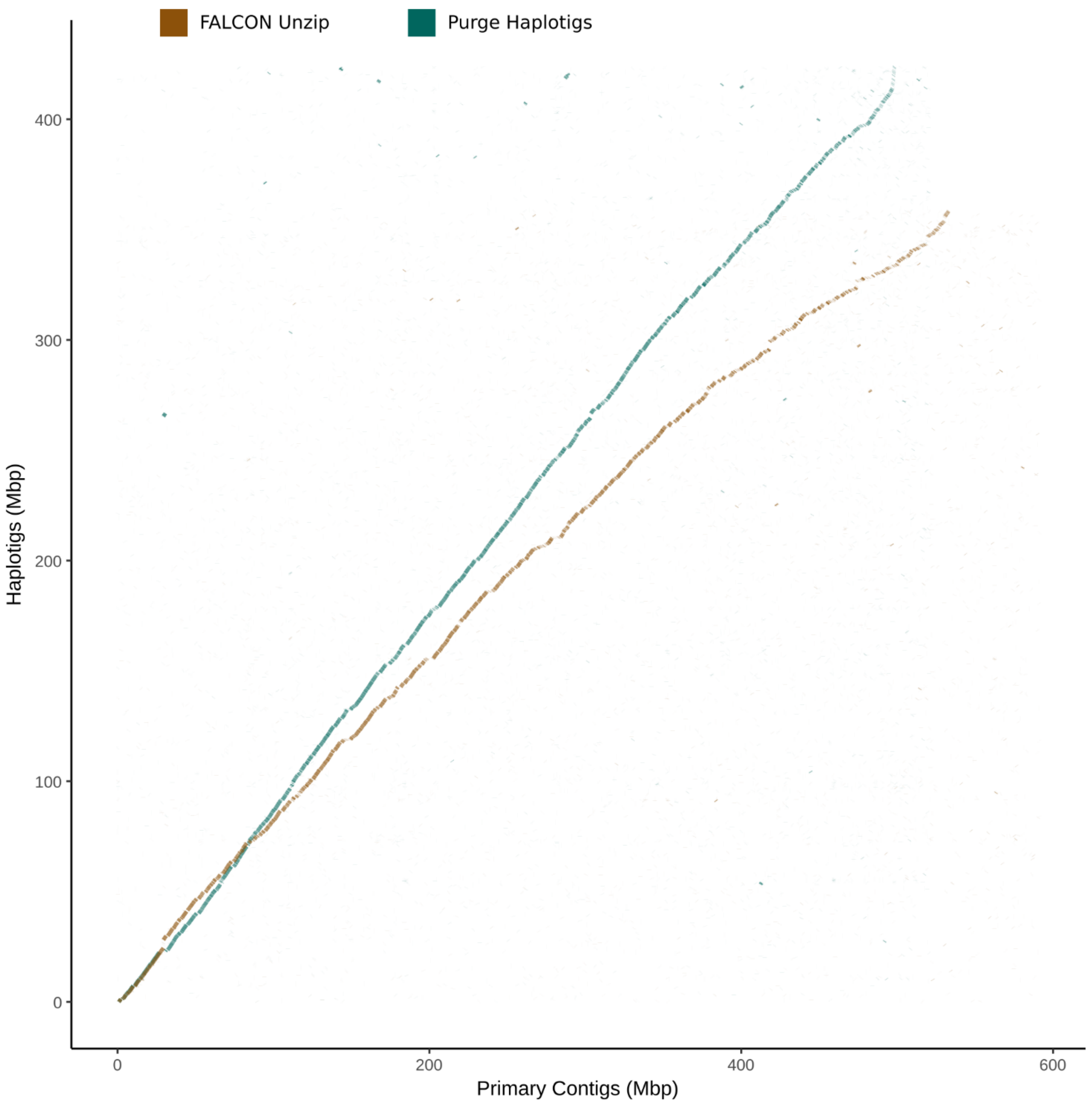

Dotplots for *Vitis vinifera L. Cv.* Cabernet Sauvignon assemblies. Haplotigs were aligned to primary contigs, total coverage of primary contigs by haplotigs was calculated, dotplots for one-to-one best alignments are shown. There was 70.8 % and 82.1 % coverage of primary contigs by haplotigs for the draft FALCON Unzip and the Purge Haplotigs-processed assemblies respectively. Vertical gaps correspond to sequence in haplotigs that is not present in the primary contigs, and horizontal gaps correspond to sequence in the primary contigs not present in the haplotigs.

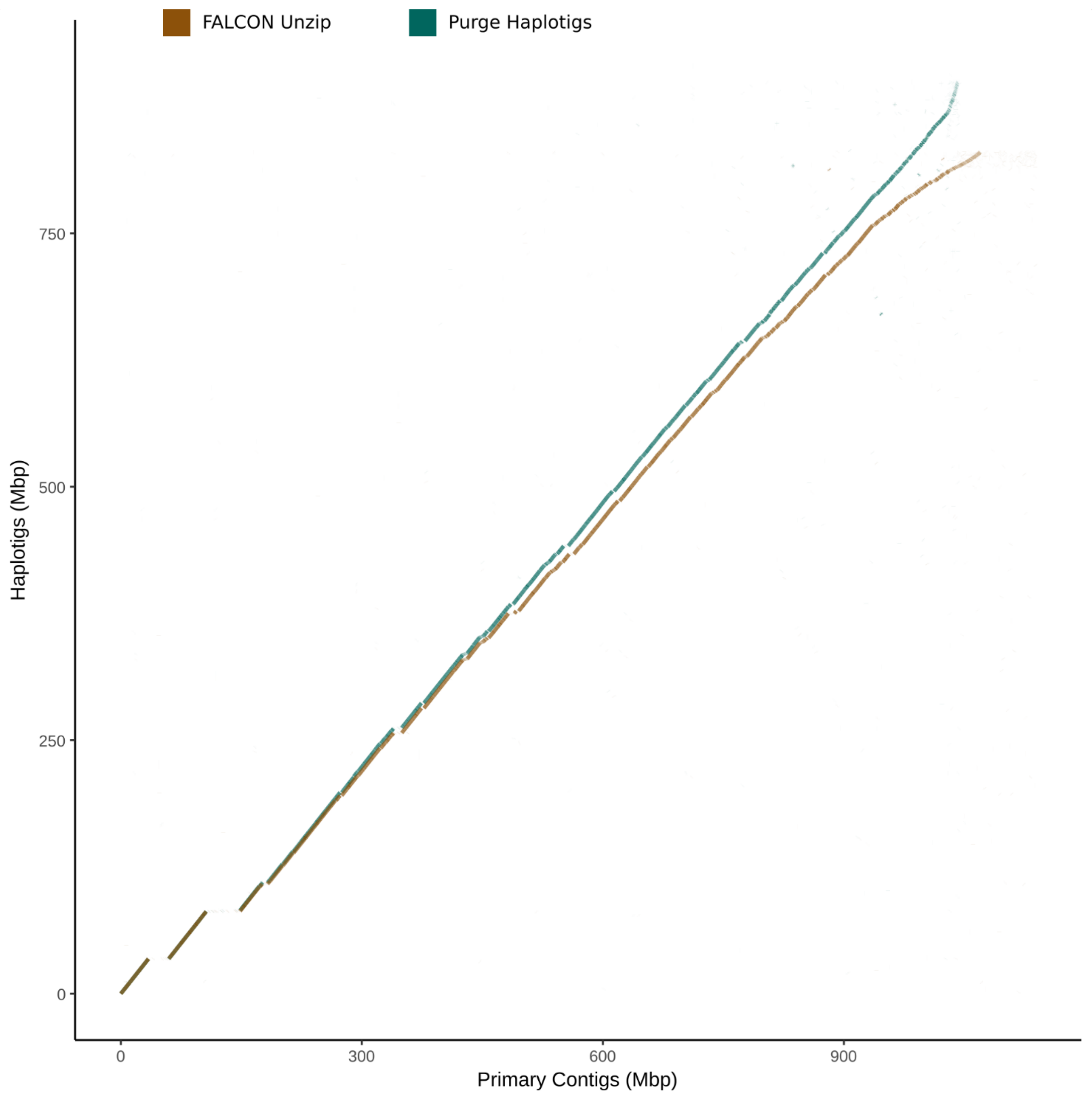

Dotplots for *Taeniopygia guttata* assemblies. Haplotigs were aligned to primary contigs, total coverage of primary contigs by haplotigs was calculated, dotplots for one-to-one best alignments are shown. There was 76.4 % and 82.0 % coverage of primary contigs by haplotigs for the draft FALCON Unzip and the Purge Haplotigs-processed assemblies respectively. Vertical gaps correspond to sequence in haplotigs that is not present in the primary contigs, and horizontal gaps correspond to sequence in the primary contigs not present in the haplotigs.
