# Supplementary material for: Purge Haplotigs: allelic contig reassignment for third-gen diploid genome assemblies
Source: BMC Bioinformatics. 2018 Nov 29;19:460. doi: 10.1186/s12859-018-2485-7 (PMC6267036; doi:10.1186/s12859-018-2485-7)
Supplement: Supplementary file 4 — Circos Plots and mapping statistics for C. pyxidata, and T. guttata. (PDF 3318 kb) [file 12859_2018_2485_MOESM4_ESM.pdf]

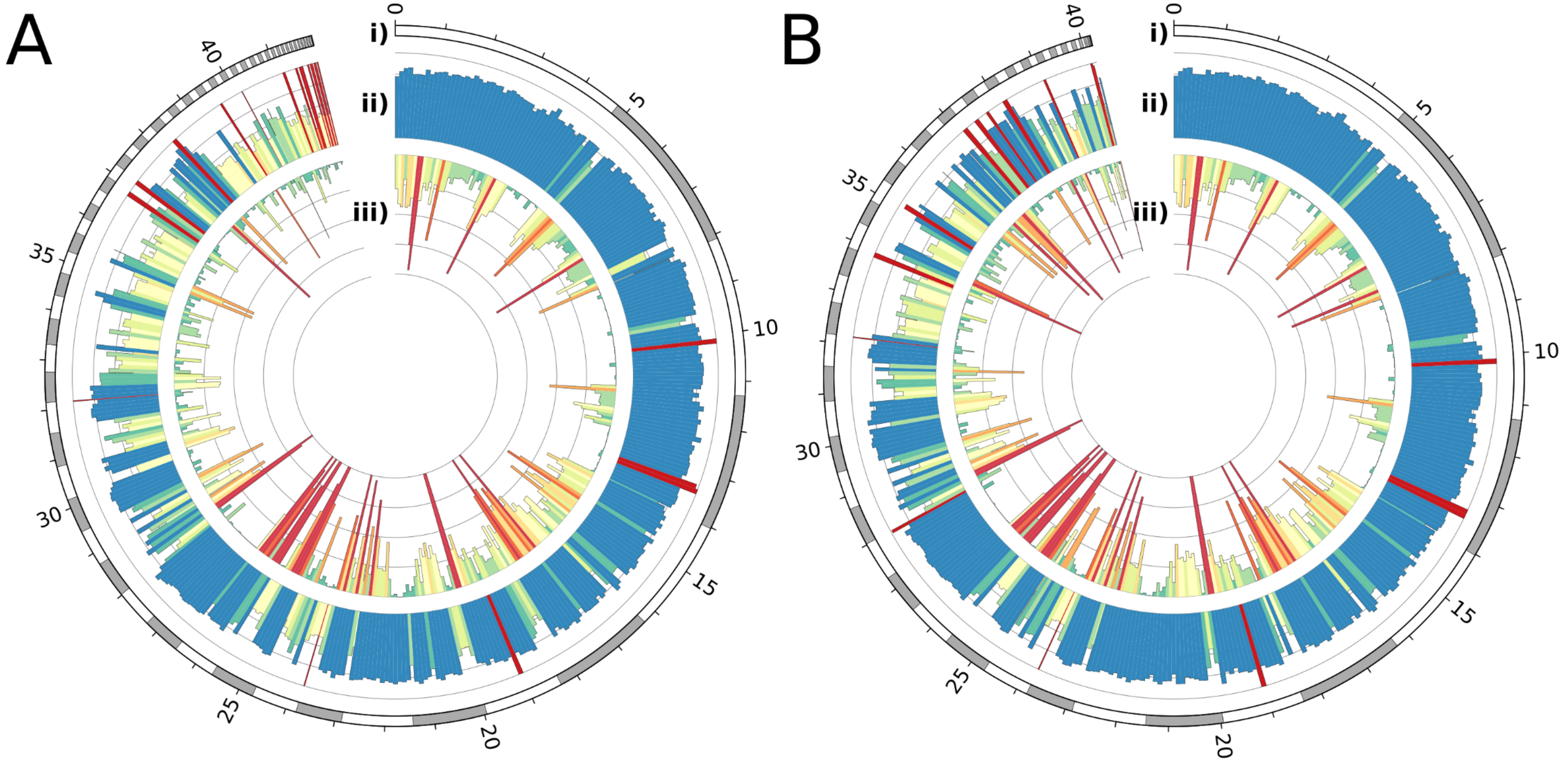

Circos plots for *Clavicorona pyxidata* haploid assemblies. Illumina PE reads were mapped, and heterozygous SNPs were called for the draft FALCON Unzip assembly (*A*) and the assembly curated with Purge Haplotigs (*B*). The tracks shown in the circos plots are: Contigs (ordered by length) (*i*), Read-depth histogram (reads per genome window; blue: median read-depth, yellow: half read-depth, red: very low/high read-depth) (*ii*), and SNP density (SNPs per genome window; blue: low SNP density, red: high SNP density) (*iii*). There were 162.5, 175.6, and 145.0 thousand filtered heterozygous SNP calls for the draft, Purge Hapltogs-processed, and Redundans-processed assemblies respectively.

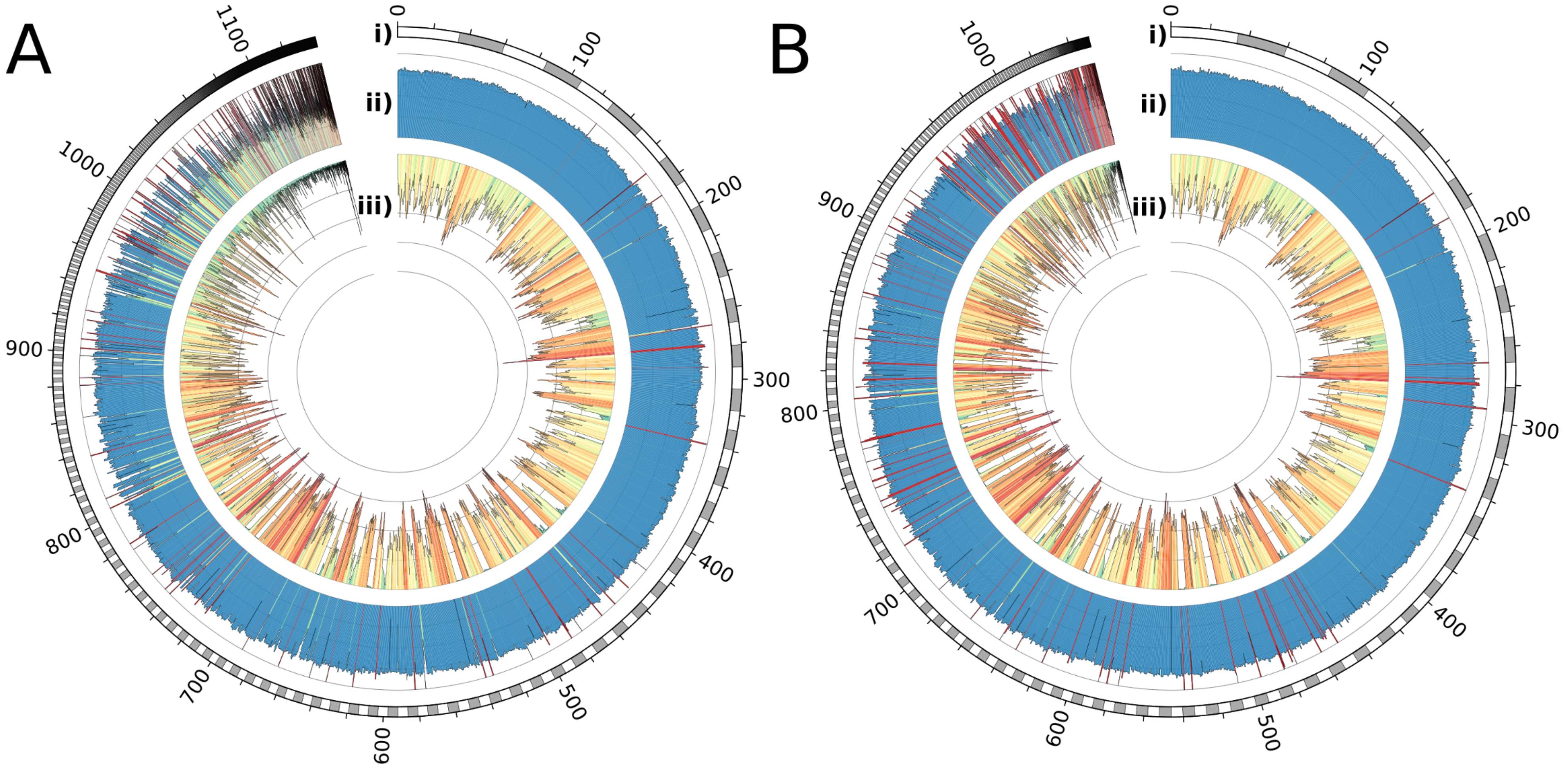

Circos plots for *Taeniopygia guttata* haploid assemblies. Illumina PE reads were mapped, and heterozygous SNPs were called for the draft FALCON Unzip assembly (*A*) and the assembly curated with Purge Haplotigs (*B*). The tracks shown in the circos plots are: Contigs (ordered by length) (*i*), Read-depth histogram (reads per genome window; blue: median read-depth, yellow: half read-depth, red: very low/high read-depth) (*ii*), and SNP density (SNPs per genome window; blue: low SNP density, red: high SNP density) (*iii*). There were 6.21, 6.38, and 6.36 million filtered heterozygous SNP calls for the draft, Purge Hapltogs-processed, and Redundans-processed assemblies respectively.
